# Extracellular vesicle-encapsulated miR-30e suppresses cholangiocarcinoma cell invasion and migration via inhibiting epithelial-mesenchymal transition

**DOI:** 10.18632/oncotarget.24711

**Published:** 2018-03-27

**Authors:** Yu Ota, Kenji Takahashi, Shin Otake, Yosui Tamaki, Mitsuyoshi Okada, Kazunobu Aso, Yuichi Makino, Satoshi Fujii, Tsuguhito Ota, Masakazu Haneda

**Affiliations:** ^1^ Division of Metabolism and Biosystemic Science, Department of Medicine, Asahikawa Medical University, Asahikawa, Hokkaido, Japan; ^2^ Department of Laboratory Medicine, Asahikawa Medical University, Asahikawa, Hokkaido, Japan

**Keywords:** extracellular vesicles, exosome, microRNA (miRNA), epithelial-mesenchymal transition (EMT), cholangiocarcinoma

## Abstract

Early-staged cholangiocarcinoma (CCA) is difficult to diagnose due to its high potential for invasion and metastasis. Epithelial-mesenchymal transition (EMT) is induced by transforming growth factor-β (TGF-β) in a process thought to be important for invasion and metastasis in several cancers, including CCA. Although microRNAs (miRNAs) have been implicated in the pathogenesis of several malignancies, their roles to CCA are not clearly understood. Some miRNAs were reported to be included in extracellular vesicles (EVs) and transferred from their donor cells to other cells, modulating recipient cell behaviors. In this study, the involvement and functional roles of EV-contained miRNAs during EMT in human CCA were determined. Expression profiling identified a subset of miRNAs that were reduced by TGF-β in CCA cells. Among these, miR-30e was highly downregulated by TGF-β and predicted to target Snail, which is an EMT-inducible transcription factor. MiR-30e overexpression suppressed cell invasion and migration via inhibiting EMT, whereas miR-30e inhibition promoted EMT, cell invasion and migration. Moreover, miR-30e was enriched in EVs derived from CCA cells after miR-30e overexpression, and miR-30e intercellular transfer through EVs suppressed EMT, cell invasion and migration in recipient CCA cells. Together, our results suggest that EV-mediated miR-30e transfer could inhibit EMT via directly targeting Snail, which subsequently suppresses CCA cell invasion and migration. These findings provide several new insights into regulatory mechanisms of tumor invasion and metastasis in human CCA.

## INTRODUCTION

Cholangiocarcinoma (CCA) is a tumor originating in the epithelium of bile ducts and classified as intrahepatic, extrahepatic or perihilar according to different anatomical locations [1]. Because of the lack of early clinical symptoms and specific biomarkers, CCA is usually diagnosed at advanced stages, for which curative surgery is not a viable option [2, 3]. Thus, there is a particular need for early diagnostic and novel therapeutic applications for CCA to improve its poor prognosis.

Epithelial-mesenchymal transition (EMT) is a process through which epithelial cells morphologically transform into cells with a mesenchymal phenotype. Tumor cells, including CCA, with epithelial characteristics exhibit significantly enhanced invasion and migration abilities after EMT [4]. EMT is promoted by transcription factors such as Snail, Slug, Twist, ZEB1 and ZEB2 [5–8], and is characterized by a reduction of epithelial markers, including E-cadherin, and the induction of mesenchymal markers such as N-cadherin and Vimentin [9]. Snail plays a critical role in EMT as a potent E-cadherin transcriptional suppressor; Snail suppression has been shown to increase E-cadherin expression and inhibit EMT [10]. Transforming growth factor-β (TGF-β) is also a strong EMT-inducer [9], and a recent study reported that human CCA cells undergo EMT via Snail activation by TGF-β [11]. Thus, EMT is an essential process for invasion and metastasis and inhibiting this pathway could improve the prognosis of CCA patients.

MicroRNAs (miRNAs) are small non-protein coding RNA (ncRNA) molecules of approximately 22 nucleotides that are known to regulate gene expression at the post-transcriptional level by either inducing target mRNA degradation or blocking translation through incomplete binding to the 3′ untranslated region (3′UTR) of target mRNAs [12]. Dysregulated miRNA expression has been reported in several human diseases including cancers and can modulate cell signaling and behaviors. Recent studies have shown that miRNAs can target various oncogenes, tumor suppressors and EMT regulators to influence tumor progression. Therefore, inhibiting EMT by modulating miRNAs might be a strategy to prevent cancer cell invasion and metastasis [13, 14]. Although the contribution of miRNAs to human liver diseases has been extensively reviewed [15], there are only a handful of studies regarding miRNAs and EMT in human CCA [16].

Extracellular vesicles (EVs) are composed of a lipid bilayer containing transmembrane proteins [17, 18] and encompass exosomes (approximately 50–150 nm in diameter), microvesicles (100–1000 nm in diameter) and apoptotic bodies (1000–5000 nm in diameter) [19]. EVs can transfer their contents such as DNA, mRNA, miRNA, proteins and lipids from donor cells to recipient cells to modulate cellular activities in recipient cells [17, 18]. It was previously reported that hepatocellular carcinoma cell-derived EVs contain miRNAs that can modulate cell behaviors in recipient cells [20]. Similar to miRNAs, cancer cell-derived EVs contain and transfer long non-coding RNA (lncRNAs). We have recently reported that hepatocellular carcinoma cell-derived EVs contain lncRNAs, such as linc-RoR and linc-VLDLR, which can be transferred to recipient cells and modulate signaling pathways related to chemotherapy or hypoxia stress resistance in recipient cells [21, 22]. These observations support a role for ncRNAs as effectors of intercellular signaling in several cancer types. Although some EV-encapsulated miRNAs have also been reported to play a crucial role in EMT and the metastatic process [23], the involvement of EV miRNAs during EMT in CCA has not been demonstrated. Therefore, our aims were to identify miRNAs that regulate invasion and metastasis by regulating the EMT pathway and to evaluate the potential roles of EV-mediated miRNA transfer in those mechanisms. Our findings provide several new insights into mechanisms of modulating EMT in CCA and the contribution of extracellular miRNA-mediated signaling to tumor cell invasion and metastasis.

## RESULTS

### TGF-β induces EMT in CCA cells

**Figure 1:**
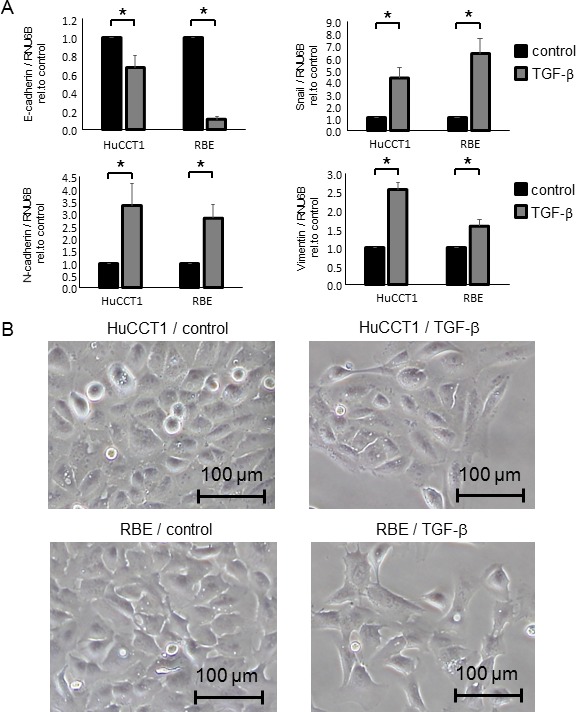
TGF-β induced EMT in CCA cells (**A**) HuCCT1 and RBE cells were treated with 10 ng/ml TGF-β for 72 h, then E-cadherin, Snail, N-cadherin and Vimentin mRNA expression was measured by qRT-PCR; relative expression normalized to RNU6B is shown. Bars represent the mean ± SEM of three separate determinants. ^*^P < 0.05. (**B**) HuCCT1 and RBE cells (1 × 10^6^ cells per 10 cm dish) were treated with 10 ng/ml TGF-β for 48 h. Representative cell morphologies are shown in the light microscope images.

Although TGF-β is a multifunctional cytokine and induces EMT in several cancer cells, it also induces cancer cell death. To assess the TGF-β concentration that induces EMT without cell death in CCA cells, we first evaluated TGF-β cytotoxicity in the human CCA cell lines, HuCCT1 and RBE. Incubation with TGF-β for 24–72 h did not affect the viability of CCA cells at doses of 0–10 ng/ml (Supplementary Figure 1). We next investigated whether incubation with 10 ng/ml TGF-β can induce EMT in CCA cells. At the molecular level, EMT is associated with the downregulation of epithelial markers and the upregulation of mesenchymal markers. We confirmed that the epithelial marker E-cadherin was decreased and that the EMT-inducible transcription factor, Snail and mesenchymal markers, N-cadherin and Vimentin were increased after 10 ng/ml TGF-β treatment in CCA cells (Figure 1A). As EMT is morphologically characterized by changes from an epithelial cell phenotype to a spindle-like appearance and functionally characterized by decreased cell adhesion, we further verified that CCA cells became spindle shaped and grew in isolation (without cell-to-cell contacts) after 10 ng/ml TGF-β treatment (Figure 1B). According to these results, we chose 10 ng/ml as the TGF-β dose for further experiments.

### MiR-30e is downregulated by TGF-β and is a candidate EMT regulator

**Figure 2:**
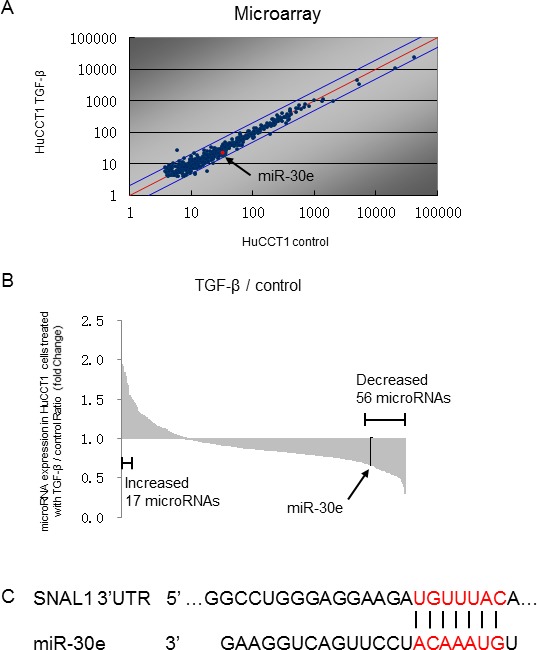
Identifying miRNAs that could regulate TGF-β-induced EMT in CCA cells HuCCT1 cells were treated with 0 (control) or 10 ng/ml TGF-β. After 72-h incubation, RNA was isolated from each experimental set of HuCCT1 cells, and expression profiling of 2555 miRNAs was performed by comparing cells with 0 and 10 ng/ml TGF-β. Expression of 451 miRNAs was detected in HuCCT1 cells. (**A**) Scatter plot of the microarray intensities of TGF-β-treated HuCCT1 cells plotted against those of control cells. (**B**) Waterfall plot showing the 56 miRNAs that were decreased by <0.67-fold and the 17 miRNAs that were increased by >1.5-fold in HuCCT1 cells treated with TGF-β. (**C**) miR-30e was predicted to target the Snail 3′UTR by TargetScan.

**Figure 3:**
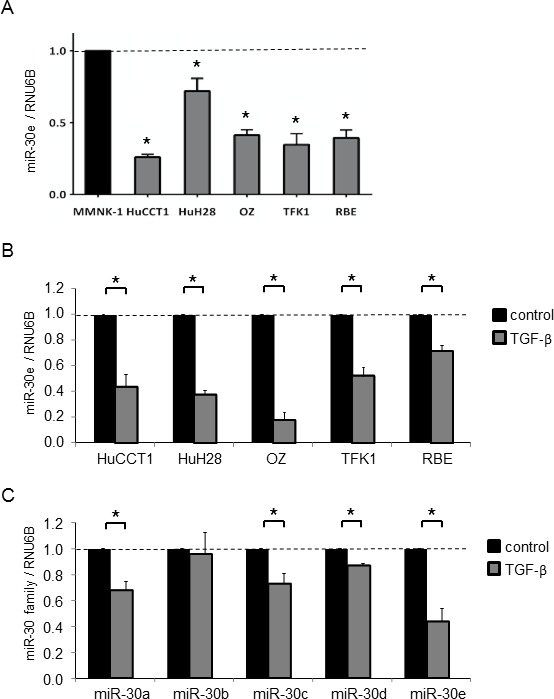
MiR-30e expression in CCA cells RNA was extracted and qRT-PCR for the miR-30 family was performed. (**A**) Basal miR-30e expression in non-malignant cholangiocytes (MMNK-1) and CCA cell lines. (**B**) miR-30e expression was assessed in CCA cell lines after incubation with 10 ng/ml TGF-β for 72 h and compared to controls. MiR-30e levels expressed relative to controls. (**C**) Expression of the miR-30 family (miR-30a, 30b, 30c, 30d and 30e) was assessed in HuCCT1 cells after incubation with 10 ng/ml TGF-β for 72 h and compared to controls. Expression of each gene was normalized to RNU6B. Bars represent the mean ± SEM of three separate determinants. ^*^P < 0.05.

We analyzed the expression of 2,555 miRNAs by microRNA arrays in CCA cells after incubation with or without TGF-β. HuCCT1 cells normally expressed 451 miRNAs, and among them, 20 were upregulated more than 1.5-fold and 56 were downregulated to less than 0.67-fold after TGF-β treatment compared with controls (Figure 2A and 2B). We focused on downregulated miRNAs, as we aimed to identify new miRNAs that could suppress TGF-β-induced EMT in CCA cells. EMT can be initiated by a group of transcription factors including Snail. Therefore, identifying factors that can suppress Snail would be important for identifying mechanisms of EMT suppression. MiR-30e was among the 56 downregulated miRNAs and was predicted to target the Snail 3′UTR by TargetScan (Figure 2C). Similar to the TargetScan results, miR-30e was also predicted to target the Snail 3′UTR by TarBase, miRNA.org, and MiRBase [24, 25]. Thus, we selected miR-30e as a candidate EMT- and tumor-suppressing miRNA. We first investigated basal miR-30e expression in several CCA cell lines and found that miR-30e expression was decreased by 0.26- to 0.72-fold in different CCA lines compared with non-malignant cholangiocytes (MMNK-1) (Figure 3A). We next examined miR-30e expression in a panel of CCA lines after TGF-β treatment. MiR-30e expression was down-regulated by TGF-β in all CCA lines (Figure 3B). The newly-identified miR-30 family is composed of miR-30a, miR-30b, miR-30c, miR-30d and miR-30e, and there have been inconsistent results regarding their function in cancer [26]. Thus, we assessed miR-30 family expression in HuCCT1 cells after incubation with TGF-β. Among the family, miR-30e expression was most significantly reduced by TGF-β treatment (Figure 3C). These results suggested that miR-30e was the most important candidate miRNA among the miR-30 family for suppressing EMT in CCA.

### MiR-30e overexpression in CCA cells inhibited TGF-β-induced EMT, invasion and migration

**Figure 4:**
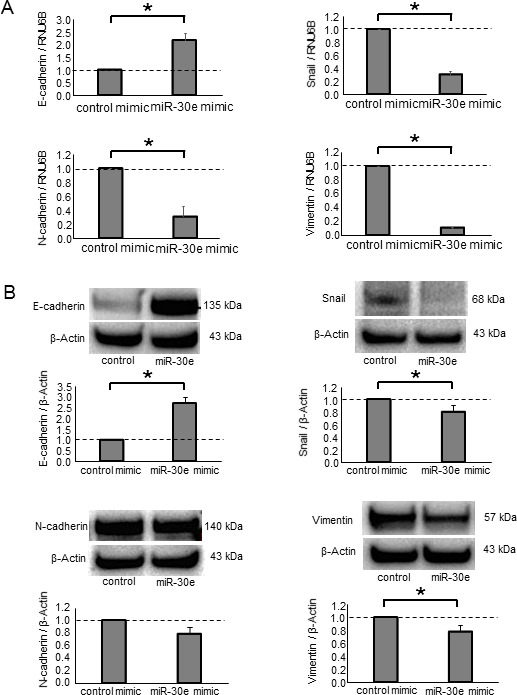

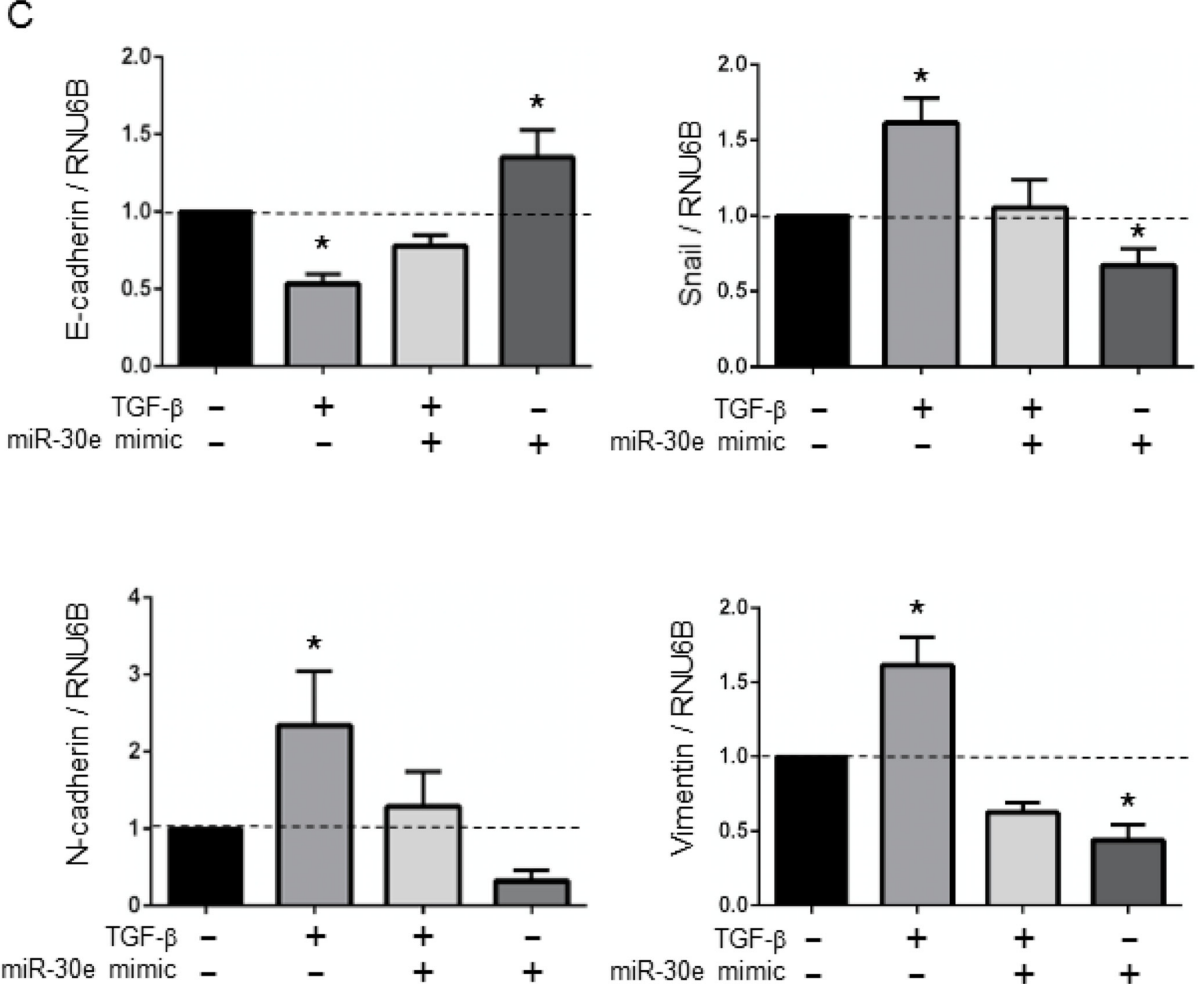
The effect of miR-30e overexpression on EMT-related genes in CCA cells (**A**, **B**) HuCCT-1 cells were transfected with 12.5 nM miR-30e or control mimic. After 48 h, RNA was isolated and qRT-PCR for E-cadherin, Snail, N-cadherin and Vimentin was performed (A). After 72 h, protein was extracted and western blots were performed using specific antibodies against E-cadherin, Snail, N-cadherin and Vimentin; relative expression normalized to β-Actin is shown (B). (**C**) HuCCT1 cells were treated with 0 or 10 ng/ml TGF-β to induce EMT. After 24 h, the cells were transfected with 12.5 nM miR-30e or control mimic. After 48 h, RNA was isolated and qRT-PCR for E-cadherin, N-cadherin, Vimentin and Snail was performed. Bars represent the mean ± SEM of three separate determinants. ^*^P < 0.05.

**Figure 5:**
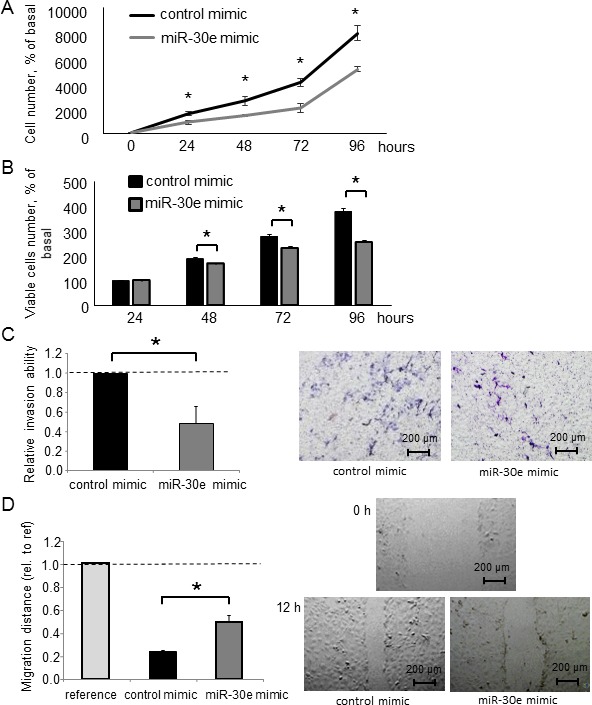
The effect of miR-30e overexpression on CCA cell phenotypes (**A**, **B**) HuCCT1 cells were transfected with 12.5 nM miR-30e or control mimic. After 24, 48, 72 and 96 h, cell proliferation was examined by cell counting using trypan blue (A) and cell viability was examined by the MTS assay (B). (**C**, **D**) HuCCT1 cells were transfected with 12.5 nM miR-30e or control mimic. After 24 h, cell invasion was assessed by the Transwell assay. Invasive cells were counted under an inverted microscope (C). After 12 h, cell migration was assessed by the wound healing assay. Wound areas were measured as described in the Experimental Procedures (D). Bars represent the mean ± SEM of three separate determinants. ^*^P < 0.05.

Having identified miR-30e as a TGF-β-regulated and candidate EMT-suppressing miRNA, we next examined the functional contribution of miR-30e to the EMT pathway. We used a miR mimic to overexpress miR-30e and confirmed its effect on miR-30e by qPCR (Supplementary Figure 2A). First, we assessed Snail mRNA expression in CCA cells transfected with either miR-30e or control mimics. Reduced Snail expression was observed with the miR-30e mimic compared with control. MiR-30e overexpression also decreased N-cadherin and Vimentin mRNA expression, whereas it increased E-cadherin expression in HuCCT1 and RBE cells (Figure 4A and Supplementary Figure 3A). Consistent with these changes, Snail, N-cadherin and Vimentin protein levels were decreased and E-cadherin was increased after miR-30e overexpression (Figure 4B and Supplementary Figure 3B). Moreover, to examine the involvement of miR-30e in TGF-β-induced EMT, we transfected miR-30e mimic into TGF-β-treated (10 ng/μl) HuCCT1 cells. TGF-β induced EMT in CCA cells, and miR-30e overexpression attenuated this effect (Figure 4C). These observations identified the EMT pathway as a downstream target of miR-30e and showed that miR-30e was an important suppressor of TGF-β-induced EMT in CCA cells. We then evaluated the effect of miR-30e overexpression on the behaviors of CCA cells. MiR-30e mimic induced decreased proliferation, viability (Figure 5A and 5B and Supplementary Figure 4A and 4B) as well as decreased cell invasion and migration (Figure 5C and 5D and Supplementary Figure 4C and 4D) in HuCCT1 and RBE cells. These results suggest that miR-30e modulates CCA cell phenotypes by regulating EMT.

### Inhibiting miR-30e in CCA cells promotes EMT, invasion and migration

**Figure 6:**
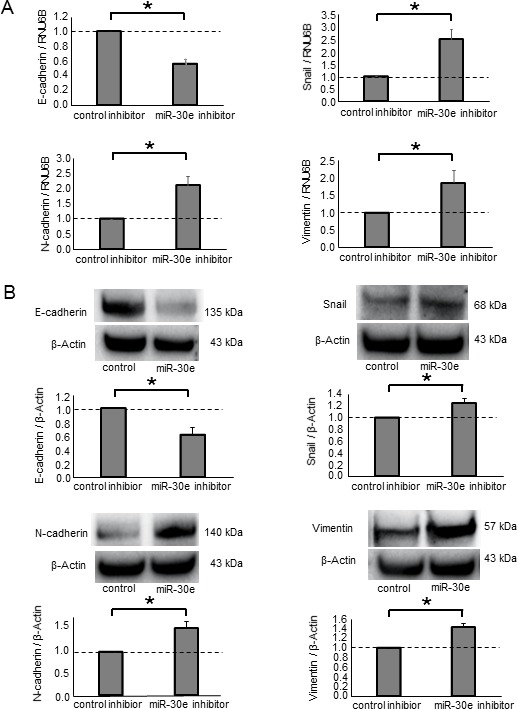
The effect of miR-30e inhibition on EMT-related genes in CCA cells (**A**, **B**) HuCCT1 cells were transfected with 25 nM miR-30e or control inhibitor. After 48 h, RNA was isolated and qRT-PCR for E-cadherin, Snail, N-cadherin and Vimentin was performed (A). After 72 h, protein was extracted and immunoblot analysis was performed using specific antibodies against E-cadherin, Snail, N-cadherin and Vimentin (B). Bars represent the mean ± SEM of three separate determinants. ^*^P < 0.05.

**Figure 7:**
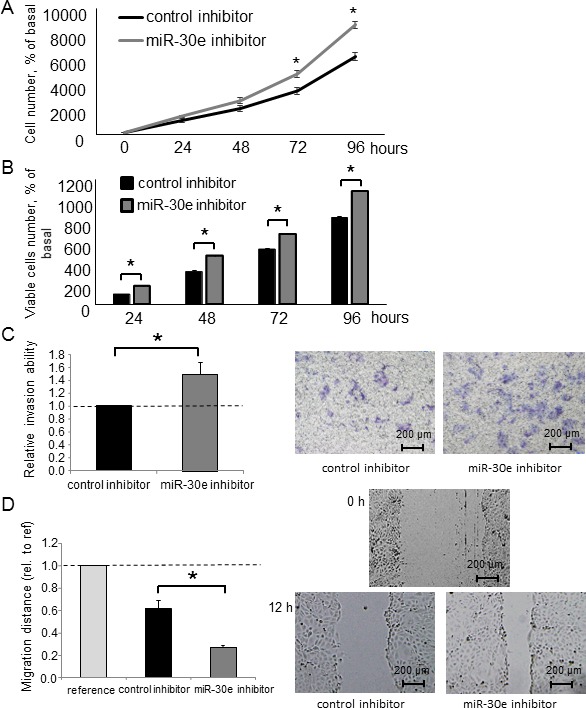
The effect of miR-30e inhibition on CCA cell phenotypes (**A**, **B**) HuCCT1 cells were transfected with 25 nM miR-30e or control inhibitor. After 24, 48, 72 and 96 h, cell proliferation was examined by cell counting using trypan blue (A) and cell viability was examined by the MTS assay (B). (**C**, **D**) HuCCT1 cells were transfected with 25 nM miR-30e or control inhibitor. After 24 h, cell invasion was assessed by the Transwell assay (C). After 12 h, cell migration was assessed by the wound healing assay (D). Bars represent the mean ± SEM of three separate determinants. ^*^P < 0.05.

To further investigate the impact of miR-30e on EMT, we used a miR-30e inhibitor to attenuate miR-30e activity in CCA cells. We analyzed the expression levels of EMT-related genes in HuCCT1 cells after incubation with the miR-30e inhibitor. Inhibiting miR-30e decreased E-cadherin expression and increased Snail, N-cadherin and Vimentin expression at both the mRNA and protein levels (Figure 6). Similar qRT-PCR and western blot results were obtained for RBE cells (Supplementary Figure 5). We next analyzed tumor cell behaviors after miR-30e inhibition. The miR-30e inhibitor significantly increased cell proliferation, viability, invasion and migration (Figure 7 and Supplementary Figure 6). These data further suggested that miR-30e regulates CCA cell phenotypes, especially invasion and migration, by suppressing EMT.

### Snail is a direct miR-30e target

**Figure 8:**
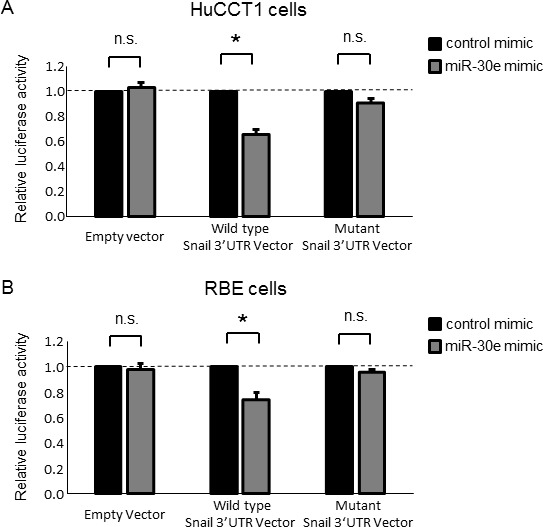
Snail is a direct target of miR-30e (**A**, **B**) HuCCT1 (A) and RBE (B) cells were transfected with 12.5 nM miR-30e or control mimic, and after 24 h, co-transfected with 2.0 μg of the Snail 3′UTR firefly luciferase reporter vector, the mutant Snail 3′UTR firefly luciferase reporter vector or empty vector and 0.1 μg of the Renilla luciferase reporter pRL-SV40. After another 24 h, relative firefly luciferase activity was measured and normalized to Renilla activity. Bars represent the mean ± SEM of three separate determinants. ^*^P < 0.05; n.s.: not significant.

The post-transcriptional regulation of gene expression by miRNAs is the result of their complementary binding to the 3′UTR of mRNA targets [27]. While bioinformatics analysis predicted that more than 500 genes could potentially be targeted by miR-30e, the Snail 3′UTR was also predicted to have miR-30e binding sites. As we initially aimed to identify miRNAs that could suppress TGF-β-induced EMT, we focused on the EMT-inducible transcription factor Snail among these potential target genes. Therefore, to confirm whether Snail is a direct miR-30e target, we co-transfected miR-30e mimic and a firefly luciferase reporter vector containing the Snail 3′UTR or a mutant construct of Snail 3′UTR into HuCCT1 and RBE cells. We found significant repression of Snail 3′UTR firefly luciferase activity in miR-30e-overexpressing cells (Figure 8), confirming the specificity of the interaction between miR-30e and the Snail 3′UTR.

### The intercellular transfer of miR-30e to CCA cells by EVs mediates invasion and migration

**Figure 9:**
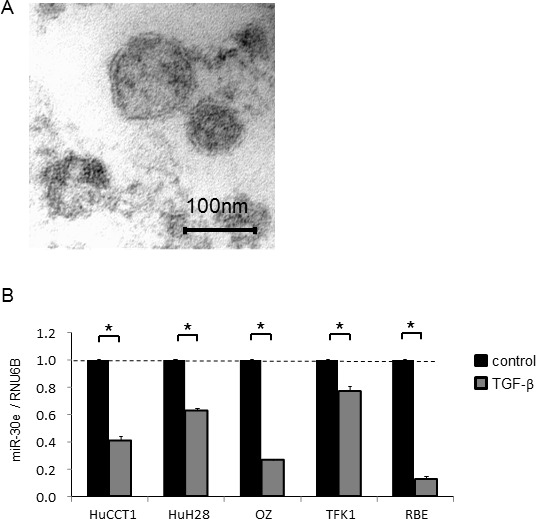

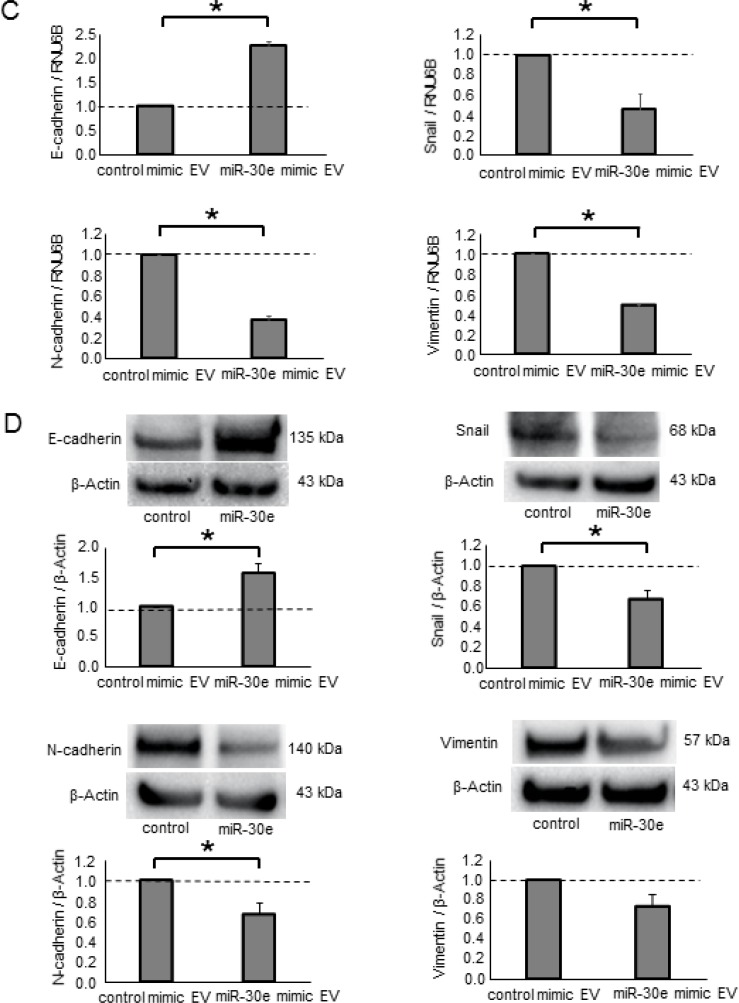
Intercellular miR-30e transfer by EVs during EMT in CCA cells CCA cells (1 × 10^6^ per 10 cm dish) were cultured for 72 h, and EVs were obtained from the conditioned medium by ultracentrifugation. (**A**) Transmission electron microscope image showing EVs isolated from HuCCT1 cells. A homogeneous population of particles was obtained. (**B**) EV RNA was isolated from CCA cells incubated with 0 or 10 ng/ml TGF-β for 72 h, and then miR-30e expression was measured by qRT-PCR. MiR-30e expression was normalized to RNU6B and expressed relative to the value in control. (**C**, **D**) HuCCT1 cells were transfected with 12.5 nM miR-30e or control mimic. After 72 h, miR-30e-enriched or control EVs were isolated. Then, 4 × 10^5^ (per well) recipient HuCCT1 cells were seeded into 6-well plates in EV-depleted medium and incubated with the miR-30e-enriched or control EVs (30 μg/ml). After 48 h, E-cadherin, Snail, N-cadherin and Vimentin expression was analyzed by qRT-PCR on RNA isolated from recipient cells (C), and by western blot using extracted protein (D). Bars represent the mean ± SEM of three separate determinants. ^*^P < 0.05.

**Figure 10:**
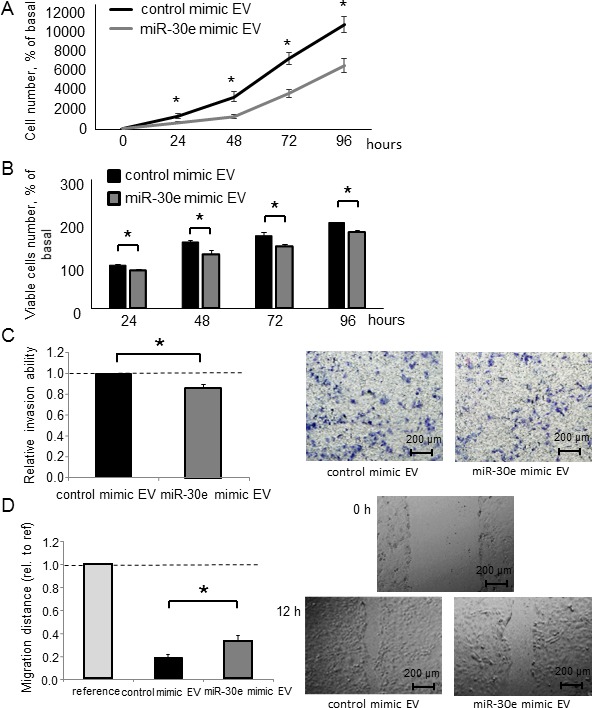
Intercellular transfer of miR-30e by EVs alters CCA cells phenotypes (**A**–**D**) HuCCT1 cells were incubated with EV solution including miR-30e-enriched or control EVs that were derived from HuCCT1 cells incubated with 12.5 nM miR-30e or control mimic (30 μg/ml) for 72 h. Cell proliferation was examined by cell counting using trypan blue (A), cell viability was examined by the MTS assay (B) after 24, 48, 72 and 96 h, cell invasion was assessed by Transwell assays after 24 h (C), and cell migration was assessed by the wound healing assay. Wounds were made after 24 h, and the wound area was measured after 12 h (D). ^*^P < 0.05.

EV contents are transferred from donor to recipient cells, and this EV-mediated crosstalk between cancer cells plays a key role in the tumor microenvironment. Thus, we sought to explore the potential of EV-encapsulated miR-30e to act as an EMT mediator in recipient cancer cells. We first isolated EVs from CCA cells by ultracentrifugation, as described in the Experimental Procedures section and visualized their morphology by electron microscopy. Consistent with previous reports, isolated EVs were approximately 100-nm microstructures with a lipid bilayer membrane (Figure 9A). Next, we verified miR-30e expression within CCA cell-derived EVs after incubation with or without TGF-β. MiR-30e expression in CCA EVs was significantly reduced by TGF-β in the same manner as in CCA cells (Figure 9B). Moreover, we confirmed markedly increased miR-30e levels in CCA cell-derived EVs after miR-30e overexpression (Supplementary Figure 2B). MiR-30e-enriched EVs were then added to recipient CCA cells. After incubation for 48 h, miR-30e expression was increased in recipient CCA cells incubated with miR-30e-enriched EVs (Supplementary Figure 2C). As miR-30e was highly expressed in EVs after miR-30e overexpression in donor cells, this indicated that miR-30e-enriched EVs could be taken up by recipient cells as a means of transferring miR-30e. Furthermore, to verify whether the transferred miR-30e could regulate EMT, we examined the expression levels of EMT-related genes in recipient cells after EV incubation. A greater increase in E-cadherin and decrease in Snail, N-cadherin and Vimentin were observed with EVs obtained from miR-30e-overexpressing cells (Figure 9C and 9D). Furthermore, incubation with miR-30e-enriched EVs significantly suppressed the proliferation, viability, invasion and migration of recipient cells (Figure 10). Taken together, these results strongly indicate that cell invasion and migration can be modulated via suppressing the EMT pathway through the intercellular transfer of miR-30e by EVs.

## DISCUSSION

Our mechanistic understanding of the metastatic cell-cell communications by cancer cell-derived EVs has recently advanced; however, little is known about the regulation of EMT through EV-encapsulated miRNAs in CCA. In this study, we showed that miR-30e could be transferred by CCA cell-derived EVs and inhibits EMT via directly targeting Snail, suppressing invasion and migration in CCA cells. This is the first report to show that EV-mediated miRNAs regulate EMT in CCA. This demonstration of EV-mediated intercellular signaling provides new insights into tumor invasion and metastasis and raises the potential for miR-30e and possibly other miRNAs to mediate biological effects in recipient cells through intercellular signaling.

EMT is the process through which epithelial cells convert into mesenchymal-like cells and plays important roles in the development of several tissues during embryogenesis. Recently, studies have demonstrated that EMT can induce cancer cell invasion and metastasis [4]. TGF-β is an EMT regulator that has been reported to induce EMT via Snail activation [9, 11]. Therefore, identifying an inhibitor of TGF-β-induced EMT in CCA cells may offer the potential to improve clinical therapies for CCA patients. In this study, we showed that TGF-β induced EMT, that miR-30e was downregulated by TGF-β, and that miR-30e was predicted by bioinformatics analyses (TargetScan, TarBase, miRNA.org and MiRBase) to directly target the Snail 3′UTR, which we confirmed in CCA cells using Luciferase reporter assay. Together, these data suggested that miR-30e contributes to altered signaling and tumor development in CCA.

The miR-30 family has been reported to suppress EMT by targeting Snail in human hepatocytes and pancreatic epithelial cells [28, 29]. Recent studies have also revealed that the miR-30 family is downregulated in tumors, where it is involved in EMT and participates in the mechanisms of tumor development and progression in several types of cancer. MiR-30a inhibits invasion and metastasis by regulating EMT through targeting Snail in non-small cell lung cancer [30]. MiR-30c inhibits EMT via targeting Slug or Snail in renal cell carcinoma and non-small cell lung cancer [31, 32]. MiR-30a, 30c and 30d also block TGF-β-induced EMT in ovarian cancer [26, 33]. Although these reports suggest that the miR-30 family works as a tumor suppressor via regulating EMT, their roles in CCA have not been fully elucidated. In this study, we identified that miR-30e could inhibit TGF-β-induced EMT, invasion and migration by targeting Snail in CCA cells. This indicated that miR-30e has the potential to be an onco-suppressor gene, similar to the other miR-30 family members. Although miR-200c and miR-214 have been reported to regulate EMT in CCA [16], our data show that miR-30e also plays a key role in regulating EMT and could be a potential therapeutic target for human CCA. As a single miRNA can target multiple mRNAs, further investigations are required to fully understand the role of miR-30e in regulating EMT.

The transfer of mRNA or ncRNA by EVs such as exosomes from cancer cells in response to environmental changes provides a potential mechanism of intercellular signaling. EVs can mediate paracrine signaling in the tumor microenvironment and play important roles in the development and progression of several cancers [18]. Additionally, increasing evidence has shown that EV-encapsulated RNA in body fluids could serve as a cancer biomarker [34]. Although some miRNAs within EVs have been reported to play important roles in regulating EMT in human lung adenocarcinoma and melanoma cells [23, 35], their roles in CCA development remain unclear. Recent experimental studies have demonstrated that miR-195 can be transferred by EVs to inhibit tumor progression in a rat model of CCA [36]. In this study, we verified that EVs can transfer miR-30e to recipient CCA cells, which inhibits EMT via directly targeting Snail in the recipient cells. One limitation of our study was the presence of various contents in EVs, rather than only transferring miR-30e in CCA-derived EVs. This raises the possibility that other EV contents potentially contributed to the observed differences in EMT, invasion and migration in response to EV. However, miR-30e could be considered as one of the important factors responsible for the observed effects, as incubation with miR-30e-enriched EVs further suppressed EMT, invasion and migration in recipient cells. The other issue raised in our study was whether extracellular miR-30e primarily exists in EVs. We have compared differential miR-30e levels from various compartments, cellular, secretary (supernatant) and EVs in isolation. We analyzed miR-30e expression by qRT-PCR using identical amounts of RNA for all reverse transcription reactions. However, there are no established housekeeping genes between cells, supernatant and EVs, making it difficult to compare mRNA levels among these different cellular compartments. Thus, the miR-30e expression results might be affected by variances in housekeeping gene Ct values. However, our preliminary results suggested that most of the extracellular miR-30e exists within EVs (data not shown). Our findings suggest that miR-30e-mediated EMT inhibition through EV communication could be a therapeutic strategy for human CCA.

In conclusion, our findings provide several new mechanistic insights into the regulation of invasion and metastasis in human CCA. The role of EV signaling in tumor cell development was evaluated, and specific EV-associated miRNA mediators such as miR-30e that are involved in modulating EMT, invasion and migration were identified. To emphasize the significance of miR-30e in CCA development, a clinical association study including an analysis of miR-30e as a serum biomarker will be needed in the future. Although further investigations will be required to fully elucidate the detailed mechanisms, novel strategies targeting EMT that use EV-encapsulated miR-30e in the tumor microenvironment may be an important therapeutic approach for advanced CCA patients.

## MATERIALS AND METHODS

### Cell lines, culture and reagents

The non-malignant human cholangiocyte cell line MMNK-1 were obtained from the JCRB Cell Bank (Osaka, Japan) and cultured as recommended by the supplier. The CCA cell lines HuCCT1, HuH28 and OZ were obtained from the JCRB Cell Bank, and TFK-1 and RBE cells were provided by the RIKEN BRC through the National Bio-Resource Project of the MEXT (Tsukuba, Japan) [37]. HuCCT1, HuH28, TFK-1 and RBE cells were cultured in RPMI 1640 medium (Thermo Fisher Scientific, Waltham, MA, USA) containing 10% fetal bovine serum (FBS) and 1% penicillin-streptomycin (Thermo Fisher Scientific). OZ cells were cultured in Williams’ medium (Thermo Fisher Scientific) with 10% FBS and 1% penicillin-streptomycin. MMNK-1 cells were cultured in DMEM high-glucose medium (Thermo Fisher Scientific) with 5% FBS and 1% penicillin-streptomycin. All cells were cultured at 37° C in a 5% CO_2_ incubator. For all EV studies, EV-depleted medium was prepared using Exosome-Depleted FBS (Thermo Fisher Scientific). TGF-β was obtained from EMD Millipore Corporation (Billerica, MA, USA). Cells were treated with 10 ng/ml TGF-β for 72 h to induce EMT.

### Isolation and characterization of EVs

Cells (1 × 10^6^) were seeded with 11 ml of EV-depleted medium in 10 cm dishes. After 3–4 d, media from 16 dishes was filtered with a filtration system (Thermo Fisher Scientific) and sequential centrifugation was performed. The media was first centrifuged at 300 × g for 10 min, then at 2000 × g for 20 min at 4° C to remove cells and cellular debris. The supernatant was then centrifuged at 10,000 × g for 70 min at 4° C to isolate EVs pellets, which were then re-suspended in 10 ml phosphate-buffered saline (PBS). All pellets resuspended in PBS were further ultracentrifuged at 100,000 × g for 70 min at 4° C. The final pellet comprised an EV preparation that contained a homogenous population of EVs and was used to isolate EV RNA or other studies, or was resuspended in 50–100 ml PBS and stored at −80° C. The protein yield was measured using a bicinchoninic acid protein assay kit (Thermo Fisher Scientific), and EV morphology was identified by transmission electron microscopy.

### RNA extraction and analysis

Total RNA was extracted from cells using the miRNeasy Mini Kit (QIAGEN, Valencia, CA, USA) or from EVs using the ExoQuick-TC^®^ (System Biosciences, Mountain View, CA, USA). For the latter, cells (1 × 10^6^) were plated with 11 ml EV-depleted medium in 10-cm dishes. After 3–4 d, the medium was collected and sequentially centrifuged at 3000 × g for 15 min to remove cells and cellular debris. The supernatant was transferred to a sterile vessel and combined with 2 ml ExoQuick-TC^®^. After an overnight precipitation at 4° C, total RNA was extracted using SeraMir™ Exosome RNA Amplification Kit (System Biosciences) according to the manufacturer’s instructions. RNA concentration was measured using a NanoDrop ND-1000 (Nano-Drop Technologies, Wilmington, DE, USA).

### MiRNA microarray analysis

RNAs from CCA cells were labeled with 3D-Gene miRNA labeling kit (Toray Industries Inc., Tokyo, Japan). Then, labeled RNAs were hybridized onto a 3D-Gene Human miRNA Oligo chip (Toray Industries Inc.). The annotation and oligonucleotide sequences of the probes conformed to the miRBase miRNA database (http://microrna.sanger.ac.uk/sequences/). After stringent washes, fluorescent signals were scanned with the 3D-Gene Scanner (Toray Industries Inc.) and analyzed using 3D-Gene Extraction software (Toray Industries Inc.). A relative expression level of a given miRNA was calculated by comparing the signal intensities of the valid spots throughout the microarray experiments. The normalized data were globally normalized per array, such that the median of the signal intensity was adjusted to 25. The microarray data can be accessed through NCBI GEO Database under NCBI accession no. GSE104629.

### Quantitative real-time PCR (qRT-PCR)

RNA was treated with RNase-Free DNase I (Qiagen) and reverse-transcribed to cDNA using the iScript^®^ cDNA Synthesis Kit (Bio-Rad Laboratories, Hercules, CA, USA). qRT-PCR was performed to detect mRNAs using an Applied Biosystems^®^ 7300 Real Time PCR System (Applied Biosystems, Foster City, CA, USA), and expression was normalized to RNU6B with SYBR green I (SYBR Advantage qPCR Premix; Clontech Laboratories, Inc., Mountain View, CA, USA). The following PCR primers were used: E-cadherin forward: 5′-TGCACCA ACCCTCATGAGTG-3′ and reverse: 5′-GTCAGTATCA GCCGCTTTCAG-3′; Snail forward: 5′-TTCTCACTGC CATGGAATTCC-3′ and reverse: 5′-GCAGAGGACAC AGAACCAGAAA-3′; N-cadherin forward: 5′-TCGCCA TCCAGACCGACCCA-3′ and reverse: 5′-TGAGGCGG GTGCTGAATTCCC-3′; Vimentin forward: 5′-CCTGAAC CTGAGGGAAACTAA-3′ and reverse: 5′-GCAGAAAG GCACTTGAAAGC-3′; RNU6B forward: 5′-CTCGCT TCGGCAGCACA-3′ and reverse: 5′-AACGCTTCA CGAATTTGCGT-3′. Expression of mature miRNAs was assessed using TaqMan human MicroRNA Assay Kit (Applied Biosystems) and normalized to RNU6B.

### Protein extraction and western blotting

Total protein was extracted from cultured cells using cOmplete^®^ Lysis-M EDTA-free and cOmplete Mini, EDTA-free, protease inhibitor cocktail tablets (Roche, Basel, Switzerland). Equivalent amounts of protein samples were mixed with NuPAGE^®^ 4× LDS Sample Buffer (Life Technologies, Grand Island, NY, USA) and separated on NuPAGE^®^ Novex 4%–12% Bis-Tris Gels (Life Technologies) and then transferred onto nitrocellulose membranes. The membranes were blocked with blocking buffer (TBST (25 mM Tris-HCl pH 7.4, 125 mM NaCl, 0.05% Tween-20) with 5% BSA (Sigma-Aldrich, St. Louis, MO, USA)) for 1 h, and then incubated overnight at 4° C with the indicated primary antibodies: rabbit monoclonal anti-E-cadherin (1:1000, Cell Signaling Technology, Danvers, MA, USA), goat polyclonal anti-Snail (1:1000, Abcam, Cambridge, UK), rabbit monoclonal anti-N-cadherin (1:1000, Cell Signaling Technology), rabbit monoclonal anti-Vimentin (1:1000, Cell Signaling Technology) and mouse monoclonal anti-β-Actin (1:5000, Santa Cruz Biotechnology, Dallas, TX, USA). Membranes were then washed three times for 15 min with TBST, and then incubated with ECL^®^ Anti-Rabbit IgG HRP-Linked Whole Antibody (1:20000, GE Healthcare, Hatfield, UK) for E-cadherin, N-cadherin and Vimentin, ECL^®^ Anti-Mouse IgG HRP-Linked Whole Antibody (1:20000, GE Healthcare) for β-Actin and ZyMax^®^ Rabbit anti-Goat IgG (H+L) HRP Conjugate (1:100000, Life Technologies) for Snail. Visualization and quantitation of protein expression were performed using ChemiDoc XRS+ Imaging System (Bio-Rad Laboratories). Relative protein expression was determined by probing the same membranes for β-Actin.

### MiRNA mimic and inhibitor transfections

CCA cells were transfected with mirVana^®^ miR-30e mimic, inhibitor or negative controls (Applied Biosystems) using Lipofectamine RNAiMAX (Life Technologies). After 48–72 h, the cells were collected and used for further experiments.

### Cell proliferation and cell viability assays

For cell proliferation studies, cells (1 × 10^4^ per well) were seeded in 24-well plates. At each time point, trypan blue staining was performed, and the number of viable cells was expressed relative to cell counts at baseline. For cell viability studies, cells (1 × 10^4^ per well) were seeded into 96-well plates. At the indicated time points, viability was assessed using CellTiter 96^®^ AQueous One Solution Assay (Promega, Madison, WI, USA). Background correction was performed by subtracting background fluorescence from wells without cells.

### Cell invasion and migration assays

Cell invasion studies were performed using 24-well Transwell^®^ permeable support 24-well plates (Corning Incorporated, Corning, NY, USA). The Transwell plates were coated with pre-diluted Matrigel (5 μg/μl; BD Biosciences, Bedford, MA, USA) prior to experimentation. Cells were seeded at a density of 5 × 10^4^ per well in 100 μl serum-free medium in the upper chamber, and the lower chamber of the Transwell was filled with 600 μl of medium supplemented with 10% FBS. After 24 h incubation, cells remaining on the upper surface of the Transwell chamber were removed with a cotton swab. The cells that had invaded through the Matrigel to the bottom of the insert were fixed and stained using Diff-Quik^®^ (Sysmex, Hyogo, Japan). The number of migrated cells was counted in three random high-powered fields under a light microscope, and the relative number was calculated. For cell migration studies, cells were seeded into 6-well plates at a density of 4 × 10^5^ cells/well and grown to 90% confluence. Straight scratches were created using a sterile pipette tip. Floating cells were removed and the cultures were maintained. Images of wounds were taken immediately and after 12 h. Cell migration was assessed by measuring the wound closure covered by cells.

### Luciferase reporter assays

The Snail 3′UTR firefly luciferase reporter vector, the mutant Snail 3′UTR firefly luciferase reporter vector and empty vector were purchased from OriGene Technologies (Rockville, MD, USA). Cells were seeded into 6-well plates at 2 × 10^5^ cells/well and treated with 12.5 nM miR-30e or negative control mimic. After 24 h, those cells were co-transfected with 2.0 μg of Snail 3′UTR firefly luciferase reporter vector, mutant Snail 3′UTR firefly luciferase reporter vector or empty vector, and 0.1 μg of Renilla luciferase reporter pRL-SV40 (Promega) using Lipofectamine 2000 (Thermo Fisher Scientific) according to the manufacturer’s protocol. After another 24 h, luciferase activity was measured using the Dual-Luciferase Reporter Assay in accordance with the manufacturer’s instructions (Promega). Relative firefly luciferase activity was normalized to Renilla luciferase activity.

### Statistical analysis

Data were expressed as the mean and standard error from at least three replicates unless otherwise noted. Comparisons between groups were performed using the two-tailed Student’s *t*-test or one-way ANOVA followed by Bonferroni post hoc test. Results were considered statistically significant when *P* < 0.05. Data were analyzed using commercial software (Prism 6; GraphPad, San Diego, CA, USA).

## SUPPLEMENTARY MATERIALS FIGURES


