# Supplementary material for: Extracellular vesicle-encapsulated miR-30e suppresses cholangiocarcinoma cell invasion and migration via inhibiting epithelial-mesenchymal transition
Source: Oncotarget. 2018 Mar 27;9(23):16400–17. doi: 10.18632/oncotarget.24711 (PMC5893249; doi:10.18632/oncotarget.24711)
Supplement: Supplementary file 1 [file oncotarget-09-16400-s001.pdf]

Extracellular vesicle-encapsulated miR-30e suppresses cholangiocarcinoma cell invasion and migration via inhibiting epithelial-mesenchymal transition

SUPPLEMENTARY MATERIALS

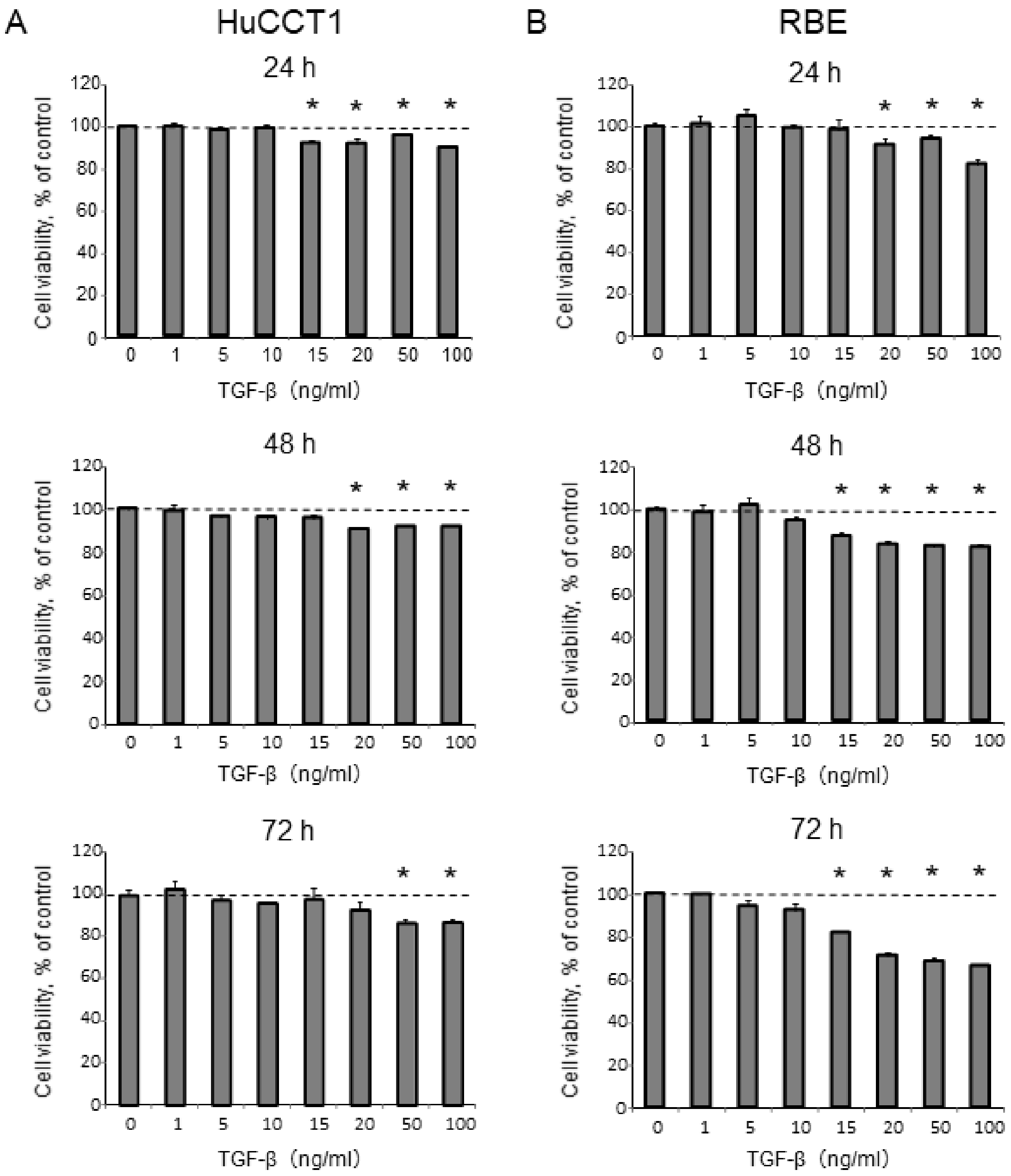

Supplementary Figure 1: The effect of TGF-β on CCA cell viability. (**A**, **B**) HuCCT1 (A) and RBE (B) cells were seeded into 96-well plates at 1×10^4 cells/well and treated with various doses of TGF-β. After 24, 48 and 72 h, cell viability was examined by the MTS assay. Bars represent the mean ± SEM of 3 separate determinants. $^*P < 0.05$.

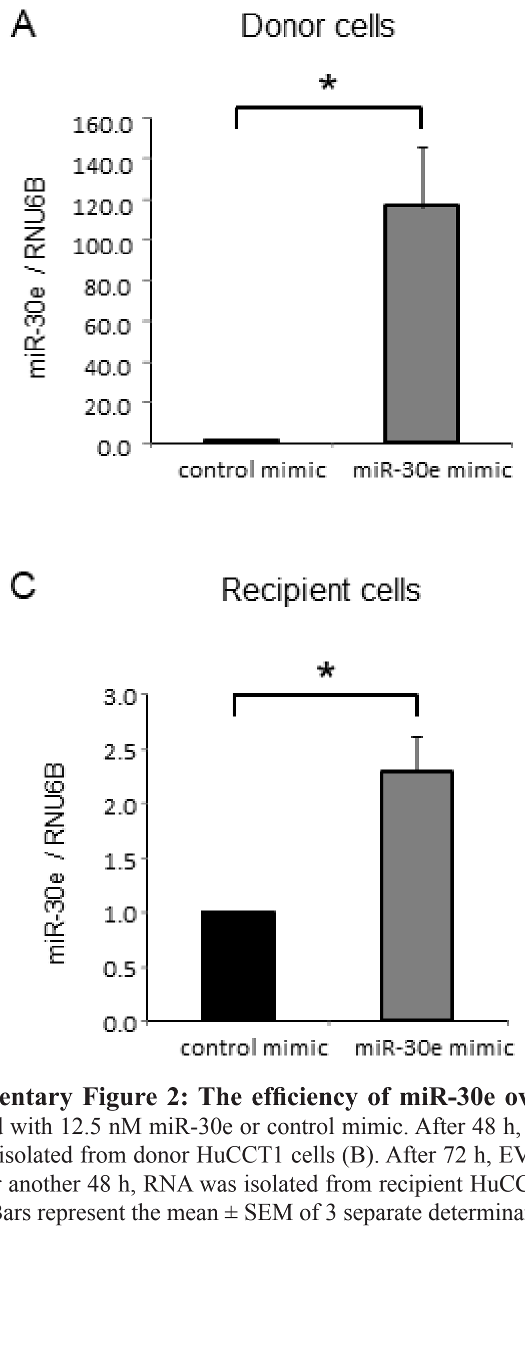

Supplementary Figure 2: The efficiency of miR-30e overexpression in CCA cells and EVs. (**A–C**) HuCCT1 cells were transfected with 12.5 nM miR-30e or control mimic. After 48 h, RNA was isolated from those donor HuCCT1 cells (A). After 72 h, EV RNA was isolated from donor HuCCT1 cells (B). After 72 h, EVs were isolated, and recipient HuCCT1 cells were incubated with those EVs. After another 48 h, RNA was isolated from recipient HuCCT1 cells (C). qRT-PCR for miR-30e was performed using the indicated samples. Bars represent the mean ± SEM of 3 separate determinants. $^{*}P < 0.05$.

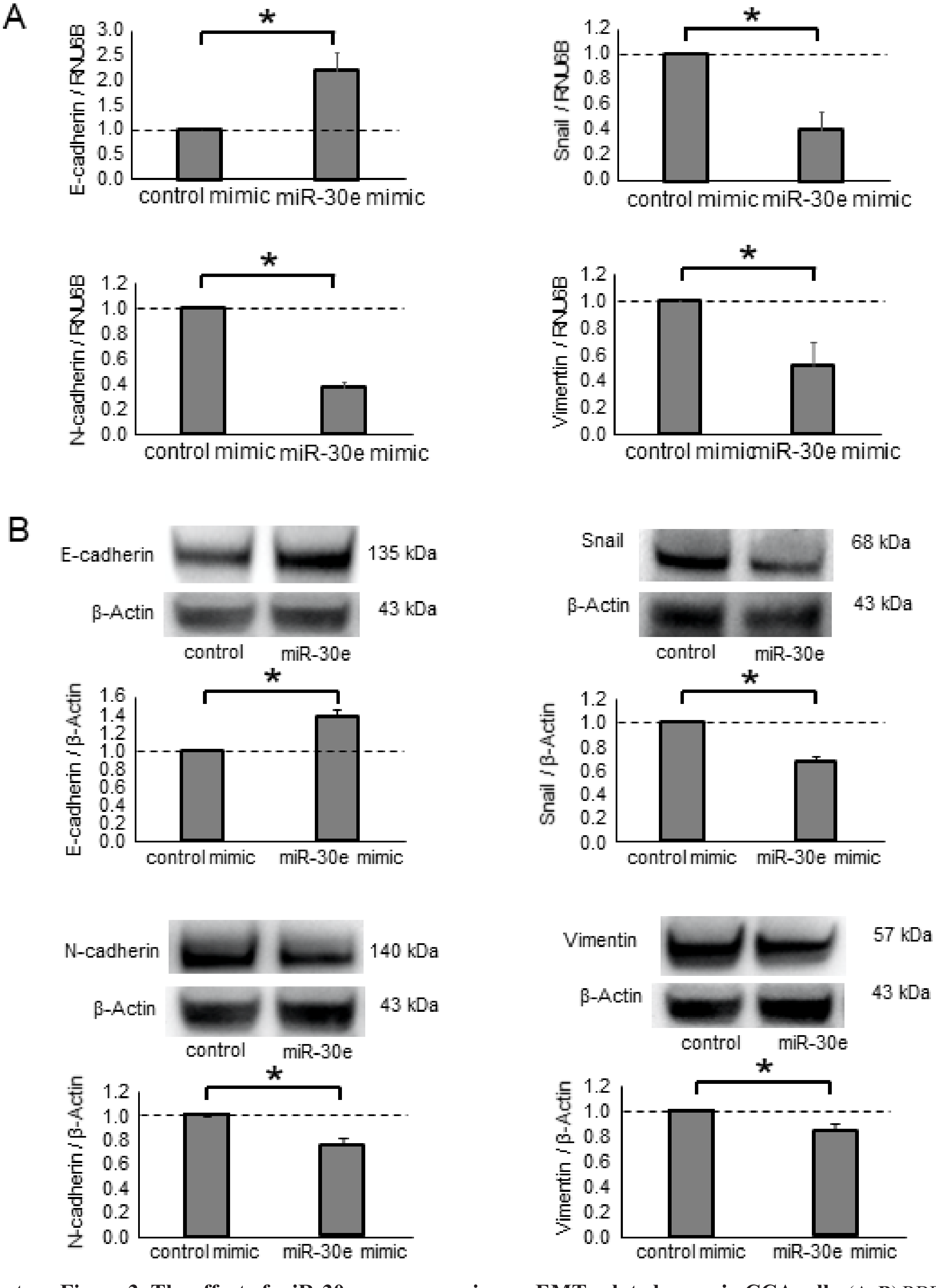

Supplementary Figure 3: The effect of miR-30e overexpression on EMT-related genes in CCA cells. (**A**, **B**) RBE cells were transfected with 12.5 nM miR-30e or control mimic. After 48 h, RNA was isolated and qRT-PCR for E-cadherin, Snail, N-cadherin and Vimentin was performed (A). After 72 h, protein was extracted and western blots were performed using antibodies specific for E-cadherin, Snail, N-cadherin and Vimentin; relative expression normalized to β-Actin is shown (B). $^{*}P < 0.05$.

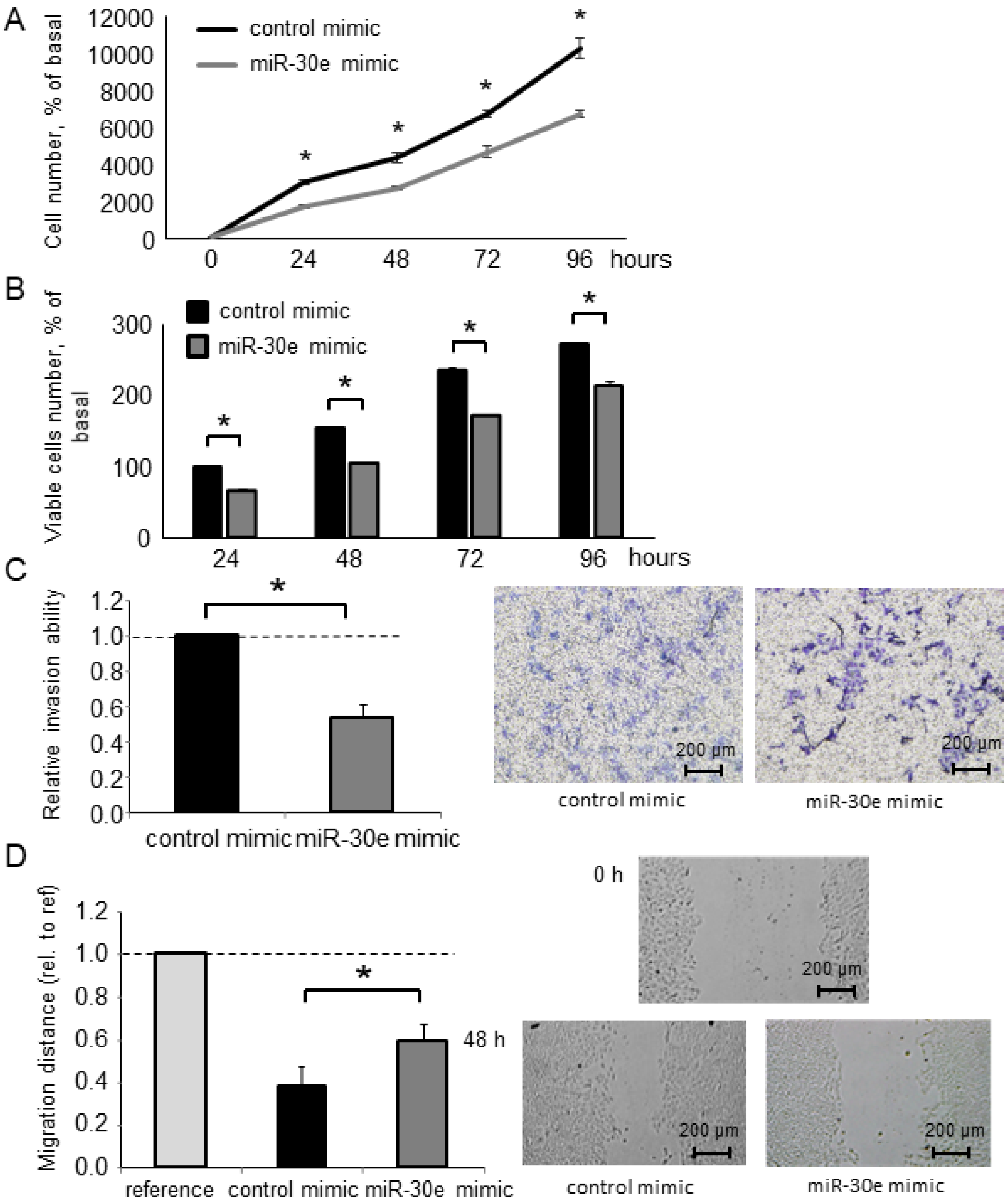

Supplementary Figure 4: The effect of miR-30e overexpression on CCA cell phenotypes. (**A**, **B**) RBE cells were transfected with 12.5 nM miR-30e or control mimic. After 24, 48, 72 and 96 h, cell proliferation was examined by cell counting using trypan blue (A), and cell viability was examined by the MTS assay (B). (**C**, **D**) RBE cells were transfected with 12.5 nM miR-30e or control mimic. After 24 h, cell invasion was assessed by the Transwell assay. Invasive cells were counted under an inverted microscope (C). After 48 h, cell migration was assessed by the wound healing assay. Wound areas were measured as described in the Experimental Procedures (D). $^{*}P < 0.05$.

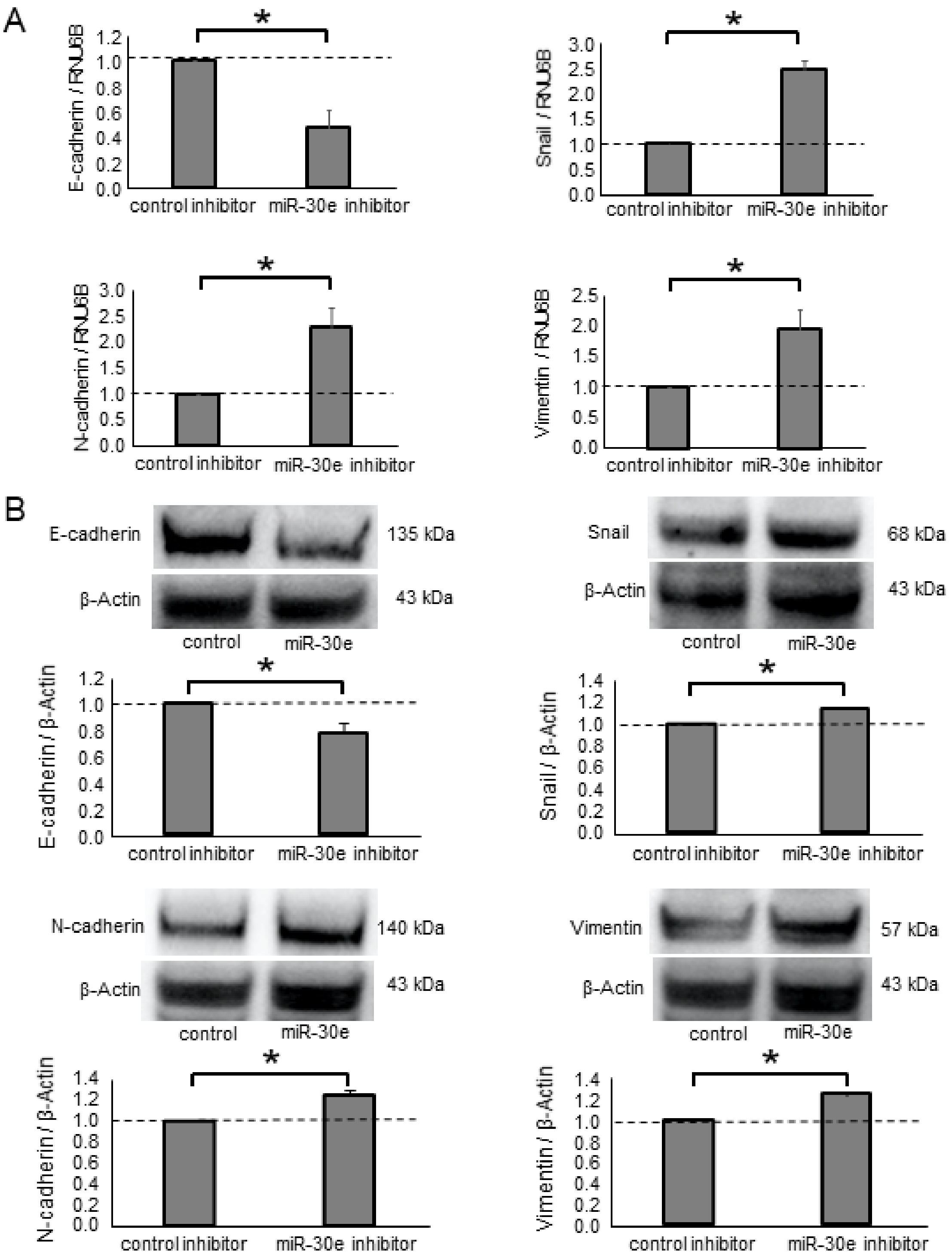

Supplementary Figure 5: The effect of miR-30e inhibition on EMT-related genes in CCA cells. (**A**, **B**) RBE cells were transfected with 25 nM miR-30e or control inhibitor. After 48 h, RNA was isolated and qRT-PCR for E-cadherin, Snail, N-cadherin and Vimentin was performed (A). After 72 h, protein was extracted and immunoblot analysis was performed using antibodies specific for E-cadherin, Snail, N-cadherin and Vimentin (B). $^{*}P < 0.05$.

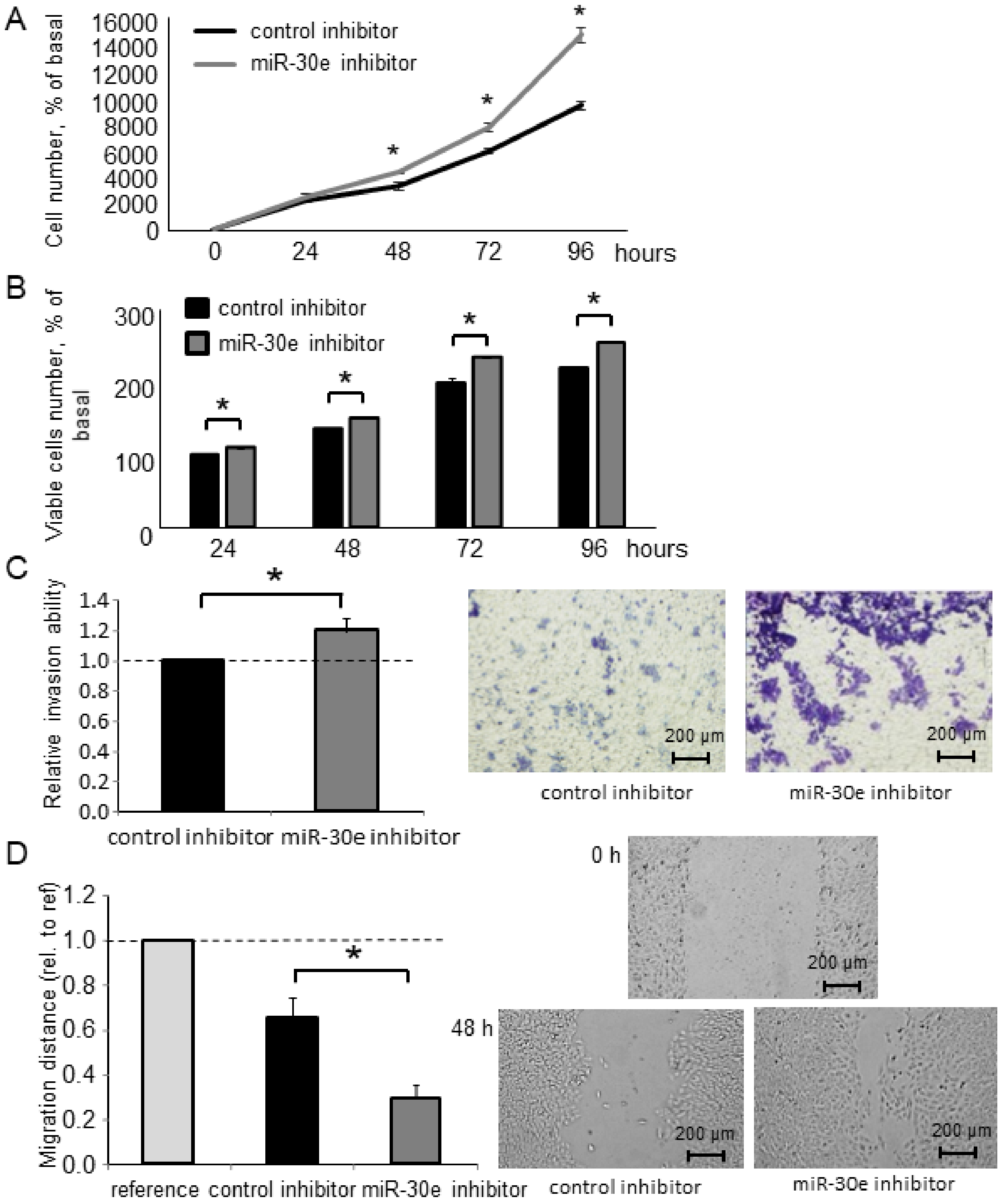

Supplementary Figure 6: The effect of miR-30e inhibition on CCA cell phenotypes. (**A**, **B**) RBE cells were transfected with 25 nM miR-30e or control inhibitor. After 24, 48, 72 and 96 h, cell proliferation was examined by cell counting using trypan blue (A), and cell viability was examined by the MTS assay (B). (**C**, **D**) RBE cells were transfected with 25 nM miR-30e or control inhibitor. After 24 h, cell invasion was assessed by the Transwell assay (C). After 48 h, cell migration was assessed by the wound healing assay (D). $^{*}P < 0.05$.
